# Efficacy and safety of Chuankezhi injection in patients with chronic obstructive pulmonary disease: A systematic review and meta-analysis protocol

**DOI:** 10.1097/MD.0000000000018620

**Published:** 2020-01-10

**Authors:** Zhihui Zhou, Wenjiang Zheng, Ting Liang, Qian Yan, Chaoyuan Zhang, Huiting Huang, Xiaohong Liu, Xiaohan Ye

**Affiliations:** a Dongguan Hospital of Traditional Chinese Medicine Affiliated to Guangzhou University of Chinese Medicine, Dongguan; b The First Clinical College of Guangzhou University of Chinese Medicine; c The First Affiliated Hospital of Guangzhou University of Chinese Medicine, Guangzhou; d Dongguan Hospital of Traditional Chinese Medicine, Dongguan, Guangdong, China.

**Keywords:** chronic obstructive pulmonary disease, Chuankezhi injection, meta-analysis, protocol, systematic review

## Abstract

**Background:**

Chuankezhi injection (CKZ) is gaining increasing popularity for chronic obstructive pulmonary disease (COPD) treatment, yet their comparative effectiveness and safety remain unclear. Therefore, we will provide a protocol to assess the efficacy and safety of CKZ for COPD.

**Methods:**

From now until June 2020, we will conduct a comprehensive and systematic literature search in 4 Chinese and 4 English databases, and the use of CKZ in the treatment of COPD will be included in randomized controlled trials, as well as all the treatment of stable COPD during the treatment of all CKZ. The risk assessment of the bias tool in Cochrane 5.1.0 will be combined with the quality of the trial. The 2 investigators will independently perform quality assessments and data extractions for the included studies in strict accordance with inclusion and exclusion criteria and perform the meta-analysis with Stata 15 software (version 15.0, StataCorp, College Station, TX).

**Results:**

Further evidence of CKZ treatment for COPD will be provided by this study.

**Conclusion:**

The efficacy and safety assessment of CKZ for COPD will be supported by this protocol.

**PROSPERO registration number:**

ROSPERO CRD 42019134133.

## 1 Introduction

Chronic obstructive pulmonary disease (COPD) is an important cause of rising rates of disease and death worldwide, which is characterized by restricted airflow and persistent respiratory symptoms.^[1]^ This situation is becoming increasingly serious. COPD is reported to have affected more than 100 million people in China, creating a serious economic and social burden.^[2,3]^ Generally, respiratory infection is the most common cause; besides, repeatedly acute attacks will accelerate the progress of the disease. Meanwhile, the current Western medicine treatment plans of COPD mainly consist of anti-infective, anti-inflammatory, dilated bronchial, and mechanical ventilation therapy. At present, the most effective anti-inflammatory drug is glucocorticoid, and patients can benefit a lot from the proper use of it. However, a number of studies have demonstrated that hormones can neither control the progression of airway inflammation in COPD treatment effectively, nor reverse the decline of lung function. Apparently, the effects of hormone therapy are not as sensitive as we expect.^[4]^ Chronic and repeated respiratory tract infections in COPD patients can lead to immune dysfunction. Immune imbalance has been considered by some studies as an important physiological basis for the continuous development of COPD.^[5]^ COPD is a disease that impairs cellular and humoral immune function in patients to varying degrees, leading to the production of pathogens. Microorganisms can easily invade the human body and cause infection, and the time is long and difficult to cure, which indicates that the immune function of the human body is closely related to the occurrence and outcome of the disease. Therefore, the curative effect of conventional Western medicine treatment still needs to be improved.

Chuankezhi injection (CKZ), composed of *Epimedium* and *Morinda*, has been widely used to treat respiratory disease in mainland China.^[6,7]^ Modern pharmacology shows that CKZ has anti-allergic and enhanced humoral and cellular immunity. Studies suggest that *Epimedium* has the ability to increase T cell proliferation and secretion of interleukin-2 and tumor necrosis factor-α, and has the ability to promote humoral and cellular immunity.^[8,9]^ Some studies observed the effects of *Epimedium* on the neuroendocrine immune function of the hypothalamic-pituitary-adrenal-thymus axis-suppressed model,^[10]^ and the results showed that *Epimedium* can improve the exogenous cortisol to the hypothalamic-pituitary-adrenal-thymus axis.^[11]^ The inhibition of morphology and function suggests that *Epimedium* plays an important role in the regulation of neuroendocrine immunity. Studies have shown that the chemical constituents of *Morinda officinalis* include polysaccharides, terpenoids, iridoids, amino acids, and trace elements, and have pharmacological effects such as anti-aging, anti-osteoporosis, and immune enhancement. Therefore, this study aims to provide sufficient evidence for the clinical application of CKZ by integrating the efficacy and safety of CKZ for COPD.

## 2 Methods

### 2.1 Study registering and reporting

The preferred reporting items for systematic review and meta-analysis (PRISMA) for reporting the results of the review will be followed by this research. PROSPERO (international register of expectations system evaluation) (CRD 42019134133) has registered this plan. The publication of the final report will record any protocol changes made during the implementation. The PRISMA-P guidelines will be followed.^[12]^ We will use the PRISMA extension statement to ensure all aspects of reporting methods and results.

### 2.2 Eligibility criteria

Using the population-intervention-comparative-results-study design framework, the eligibility criteria for the review are as follows.

#### 2.2.1 Population

Regardless of age, gender, ethnicity, educational or economic status, and whether they were outpatients or inpatients, the study will be based on patients diagnosed with COPD.^[13]^ Most of the researches that were published in China in 2017 will use the revised standards of the Chinese COPD experts’ consensus.

#### 2.2.2 Interventions/comparators

The intervention of the trial group: CKZ combined with Western medicine. The intervention of the control group: Western medicine alone. Conventional treatment refers to Western medicine standardized treatment, including oxygen, antispasmodic, antiasthmatic, anti-infective, phlegm, and nutritional support. CKZ medication methods include acupoint injection, intramuscular injection, aerosol inhalation, and so on.

#### 2.2.3 Outcome measures

Main outcomes: Lung function especially the forced expiratory volume in one second and exacerbations.^[14]^ Additional outcomes: Total effective rate and adverse reaction.

#### 2.2.4 Study design

To limit heterogeneity and enhance clinical applicability, we have defined strict inclusion/exclusion criteria for this study. We analyzed only the randomized controlled trials that were associated with CKZ in COPD treatment. We will exclude repeated studies that do not have sufficient information to compute an effect evaluation. No language or other restrictions will be applied.

### 2.3 Search strategy

We will start with the database: first, searching 4 Chinese databases (Wanfang database, China National Knowledge Infrastructure database, SinoMed, and Chongqing VIP information) and 4 English databases (Embase, Cochrane Library, Medline, and Web of Science) to ensure that all possible studies on COPD and CKZ are included. We will conduct a targeted gray literature search using clinicaltrials.gov and International Clinical Trials Registry Platform search portal to identify trials that are ongoing or have been completed.^[15]^ Lung disease experts and information experts have provided help and guidance during literature retrieval. In order to fully retrieve eligible studies, a comprehensive search strategy will be used, combining text words, Medical Subject Headings (MeSH) terms, synonyms, and headings/summary. To enhance the search strategy by several combinations, the Boolean operators “AND” and “OR” were used. Bibliographic studies were conducted in June 2019. We proceed on the basis of previous systematic evaluations in this area.^[16,17]^ Language is unrestricted in all database searches. Yet, if we search the literature, those articles that are not in Chinese or English will be excluded. Medline's search strategy is as follows:

#1Search (“Pulmonary Disease, Chronic Obstructive”[Mesh]) OR ((“COPD” or “Chronic Obstructive Pulmonary Disease” or “COAD” or “Chronic Obstructive Airway Disease” or “Chronic Obstructive Lung Disease” or “Airflow Obstruction, Chronic” or “Airflow Obstructions, Chronic” or “Chronic Airflow Obstructions” or “Chronic Airflow Obstruction”))
#2Search (“Medicine, Chinese Traditional”[Mesh]) OR ((“Traditional Chinese Medicine” or “Chung I Hsueh” or “Hsueh, Chung” or “Traditional Medicine, Chinese” or “Zhong Yi Xue” or “Chinese Traditional Medicine” or “Chinese Medicine, Traditional” or “Chinese patent medicine” or “Chinese patent drug” or “proprietary Chinese medicine” or “proprietary Chinese drug” or “Chinese herbal injection” or “Chinese medicine injection”))
#3 Search (“Injections” [Mesh]) OR ((“Injection” or “Injectables” or “Injectable”))
#4Search (“Chuankezhi”)
#5 #3 AND #4
#5 #1 AND #2 AND #5

### 2.4 Study selection and data extraction

To include studies that meet the criteria, 2 researchers will use EndNote X9.0 to read the preliminary summary of articles obtained from the above database; then, we will scan the full text for a second analysis to determine the final qualification. We resolved the differences through discussion or with the assistance of a third investigator. We will use Microsoft Excel 2017 to collect relevant information and extract data from it. We will divide the extracted information into 5 parts:

1. First author, published information: publication year, country of publication and journal;
2. General information of patients: illness, gender, age, sample capacity, qualification standards, numbers of dropouts, and baseline information;
3. The specific details of the intervention and control that we take: treatment course, dosages, and drugs;
4. Outcome indicators: lung function especially the forced expiratory volume in one second, exacerbations, total effective rate, adverse reaction, etc^[18]^; and
5. Adverse events and risk of bias assessment.

### 2.5 Quality assessment

The Risk of Bias Tool in Cochrane Handbook 5.1.0 will be used to assess the risk of bias. The second reviewer will independently assess the risk of bias in a quarter of the selected study and will first resolve the differences through discussion and then in consultation with the third reviewer. Once this process is completed, the evaluator will assess the risk of bias in the remaining studies. We will use the alternative regression test as evidence to examine publication bias.^[19]^

Assessing sources of bias includes whether random sequence generation was used, whether treatment assignments were hidden, whether participants and researchers were unaware of the treatment participants received, the incompleteness of any major outcomes, and the selectivity of reporting. We will assess the source of each bias in each trial and classify it as “high risk,” “low risk,” or “uncertain risk.” It will describe the percentage of each category in each bias source and explain the results taking into account the risk of bias. We will also conduct sensitivity analysis, but exclude studies that report a high risk of bias in any area being analyzed. We will use the Grading of Recommendation, Assessment, Development, and Evaluation tool to evaluate the level of recommendation strength and quality of evidence.^[20]^ This assessment is based on such considerations as study design, inconsistency, methodological limitations, indirectness, imprecision, etc. The quality of evidence is classified as high, medium, low, or very low.

### 2.6 Statistical analysis

We will complete the meta-analysis using Stata 15 software (version 15.0, StataCorp, College Station, TX). To evaluate the extracted data, the weighted mean difference will be used for continuous variables. For dichotomous variables, we will apply the odds ratio to analyze. The confidence interval for continuous and binary variables will be set to 95%. Heterogeneity will be calculated based on the χ^2^ test, and the degree of heterogeneity will be determined by the values of *I*^2^ (*I*^2^ > 50% or not) or *P* value (*P *<* *.05 or not). If *I*^2^ < 50% and *P* > .05, then heterogeneity is irrelevant and a meta-analysis will be performed using the Mantel–Haenszel fixed model. If *I*^2^ ≥ 50% and *P* ≤ .05, heterogeneity needs to be analyzed. Statistical heterogeneity includes statistical heterogeneity, methodological heterogeneity, and clinical heterogeneity. We will use subgroup and sensitivity analyses to explore the sources of heterogeneity. We will use stochastic effects models for statistical heterogeneity. If there is clinical and methodological heterogeneity, we will perform a subgroup analysis. If the source of heterogeneity cannot be known, synthetic analysis will be abandoned and descriptive analysis will be adopted. We will combine the robustness of the results with the sensitivity analysis. We will use sensitivity analysis to combine the robustness of the results. We will establish a funnel plot to assess report bias in included studies. Egger test will be used to evaluate funnel plot symmetry and interpret *P* < .1 as statistically significant.^[21]^

### 2.7 Patient and public involvement

This part is not covered in this study.

### 2.8 Ethical approval

The ethical approval was not necessary because no patient and public was involved in this study.

## 3 Discussion

COPD is a progressive disease in which lung function is reduced and symptoms worsen. The most troublesome is acute exacerbation, where symptoms suddenly worsen, such as a severe cough, chest congestion, and shortness of breath, requiring urgent treatment.^[22]^ Bronchodilators, antimuscarinic agents, glucocorticoids, and anti-inflammatory agents are currently considered to be commonly used in the treatment of COPD, relieving clinical symptoms, reducing the severity and frequency of acute exacerbations, and improving exercise health and tolerance. Even though Western medicine has positive therapeutic effects for COPD, there are some serious side effects, such as somatic tremor, arrhythmia, steroid myopathy, renal impairment, etc.^[23,24]^ As a result, it is necessary to discuss a protocol that CKZ has higher therapeutic effects on COPD with few side effects. According to literature reports, effective components in CKZ can anti-allergic, enhanced humoral immunity and cellular immunity to improve COPD.

The design of this protocol conforms to the guideline of meta-analysis protocols.^[25]^ It will perform a meta-analysis based on the PRISMA extension statement. After completion, the results of the meta-analysis will be submitted to a peer-reviewed journal. Evidence of the efficacy and safety of CKZ for COPD will be provided by this protocol. From another point of view, this shows that the treatment effect, clinical treatment results, side effects, and adverse reactions have changed. The systematic review will be able to assist clinicians in making CKZ-related decisions and will be beneficial to patients with COPD.

## Author contributions

All authors provided feedback and approved the final manuscript.

**Data curation:** Zhihui Zhou, Tianqi Gao, Qian Yan.

**Formal analysis:** Wenjiang Zheng, Zhihui Zhou.

**Investigation:** Yu Hong, Wenjiang Zheng.

**Methodology:** Wenjiang Zheng, Zhihui Zhou, Huili Liao.

**Project administration:** Wenjiang Zheng, Huiting Huang, Xiaohong Liu.

**Resources:** Wenjiang Zheng, Zhihui Zhou.

**Supervision:** Huiting Huang.

**Writing – original draft:** Wenjiang Zheng, Zhihui Zhou, Tianqi Gao, Qian Yan.

**Writing – review & editing:** Wenjiang Zheng, Huiting Huang.
